# Development and validation of a sensitive sandwich ELISA against human PINK1

**DOI:** 10.1080/15548627.2025.2457915

**Published:** 2025-02-06

**Authors:** Zahra Baninameh, Jens O. Watzlawik, Bernardo A. Bustillos, Gabriella Fiorino, Tingxiang Yan, Szymon L. Lewicki, Haonan Zhang, Dennis W. Dickson, Joanna Siuda, Zbigniew K. Wszolek, Wolfdieter Springer, Fabienne C. Fiesel

**Affiliations:** a Department of Neuroscience, Mayo Clinic, Jacksonville, FL, USA; b Mayo Graduate School of Biomedical Sciences, Mayo Clinic, Jacksonville, FL, USA; c Department of Neurology, Faculty of Medical Sciences in Katowice, Medical University of Silesia, Katowice, Poland; d Department of Neurology, Mayo Clinic, Jacksonville, FL, USA

**Keywords:** Autophagy, mitophagy, P-S65-Ub, Parkin, PINK1, ubiquitin

## Abstract

The ubiquitin kinase and ligase PINK1 and PRKN together label damaged mitochondria for their elimination in lysosomes by selective autophagy (mitophagy). This cytoprotective quality control pathway is genetically linked to familial Parkinson disease but is also altered during aging and in other neurodegenerative disorders. However, the molecular mechanisms of these mitophagy changes remain uncertain. In healthy mitochondria, PINK1 protein is continuously imported, cleaved, and degraded, but swiftly accumulates on damaged mitochondria, where it triggers the activation of the mitophagy pathway by phosphorylating its substrates ubiquitin and PRKN. Levels of PINK1 protein can therefore be used as a proxy for mitochondrial damage and mitophagy initiation. However, validated methodologies to sensitively detect and quantify PINK1 protein are currently not available. Here, we describe the development and thorough validation of a novel immunoassay to measure human PINK1 on the Meso Scale Discovery platform. The final assay showed excellent linearity, parallelism, and sensitivity. Even in the absence of mitochondrial stress (i.e. at basal conditions), when PINK1 protein is usually not detectable by immunoblotting, significant differences were obtained when comparing samples from patient fibroblasts or differentiated neurons with and without PINK1 expression. Of note, PINK1 protein levels were found increased in human postmortem brain with normal aging, but not in brains with Alzheimer disease, suggesting that indeed different molecular mechanisms are at play. In summary, we have developed a novel sensitive PINK1 immunoassay that will complement other efforts to decipher the roles and biomarker potential of the PINK1-PRKN mitophagy pathway in the physiological and pathological context. **Abbreviations**: AD: Alzheimer disease; CCCP: carbonyl cyanide 3-chlorophenylhydrazone; ECL: electrochemiluminescence; ELISA: enzyme-linked immunosorbent assay; iPSC: induced pluripotent stem cell; KO: knockout; LLOQ: lower limit of quantification; MSD: Meso Scale Discovery; PD: Parkinson disease; p-S65-Ub: serine-65 phosphorylated ubiquitin; Ub: ubiquitin; ULOQ: upper limit of quantification; WT: wild-type.

## Introduction

The mitochondrial kinase PINK1 and the E3 ubiquitin (Ub) ligase PRKN together mediate the elimination of damaged mitochondria by autophagy. While both are broadly expressed and thought to safeguard mitochondrial health in a variety of cells and tissue, their loss of activity or loss of protein is specifically linked to early-onset Parkinson disease (PD) [1]. PD is a debilitating disease that is characterized by a range of motor and non-motor symptoms caused by the degeneration of dopaminergic neurons in the substantia nigra. Currently there is no cure or therapy to stop or reverse the progression of this debilitating disease.

PINK1 is a 581 amino acid protein with a molecular mass of 63 kDa [2]. It consists of an N-terminal mitochondrial targeting sequence/MTS, a transmembrane sequence/TMS, the kinase domain comprising an *N*- (residues 156–320) and C-lobe (residues 321–511) and a C-terminal region/CTR. The latter has been suggested to regulate the kinase activity [3]. Under healthy conditions, PINK1 protein levels are kept in check by proteasomal degradation [4]. PINK1 protein is imported and cleaved by PMPC (peptidase, mitochondrial processing) and PARL (presenilin associated rhomboid like) protease between Ala103 and Phe104 [5,6]. Cleaved PINK1 (now 52 kDa) then transfers back to the cytoplasm where it is rapidly degraded by the proteasome [7,8]. The truncated form of PINK1 can experimentally be stabilized by proteasome inhibitors, such as epoxomicin [9]. The disruption of the electrochemical gradient across the inner mitochondrial membrane upon damage of mitochondria prevents the import of PINK1 into the inner mitochondrial membrane and results in the lateral insertion into the outer mitochondrial membrane [10]. This results in the accumulation of the full-length protein, followed by dimerization and autophosphorylation at Ser228 with the kinase domain facing the cytoplasm [11–13].

Activated PINK1 phosphorylates ubiquitin at Ser65 (p-S65-Ub), which acts as the allosteric activator for the cytosolic E3 Ub ligase PRKN [14–16]. Recruited PRKN is also phosphorylated by PINK1, and this fully activates the E3 ubiquitin ligase functions [17–19]. Once unleashed, active PRKN ligates more Ub to mitochondrial substrates, that can then be used as substrate for the PINK1 kinase, leading to an efficient amplification of the signal [15,16,20,21]. With p-S65-Ub decorated mitochondria are recognized by autophagy adapters that facilitate the engulfment of the mitochondria into autolysosomes and their degradation in lysosomes [22–25]. Most of PD-associated mutations are located in the kinase domain of PINK1 and cause a loss of kinase function. However, some mutations have also been shown to interfere with the stabilization of PINK1. For instance, compared to wild-type (WT) PINK1, both PINK1^Q456X^ and PINK1^I368N^ lead to greatly reduced PINK1 protein levels upon treatment with mitochondrial depolarizers [26,27].

We have previously developed p-S65-Ub antibodies that can be used for immunohistochemistry and have established a sensitive p-S65-Ub ELISA as a surrogate for PINK1-PRKN mitophagy [28,29]. Data from postmortem human brain shows that the mitophagy marker p-S65-Ub is generally upregulated with age and with disease [28,30–32]. In some cases, it remains unclear, however, if the observed accumulation of p-S65-Ub is caused by increased mitochondrial damage or by reduced lysosomal degradation, both of which have been associated with physiological aging as well as different neurodegenerative conditions. Additional readouts are needed to determine the cause of increased p-S65-Ub in human brain.

As the most upstream component of the PINK1-PRKN mitophagy pathway, PINK1 protein levels and activation are a marker of mitochondrial damage. However, there are currently no validated quantitative methods to measure the abundance of this kinase. In addition, existing methods lack sensitivity to faithfully detect PINK1 in the absence of exogenous stress. Here, we have developed a specific and highly sensitive sandwich ELISA on a Meso Scale Discovery (MSD) platform to detect levels of human PINK1 protein in healthy and damaged conditions including from patient fibroblasts, isogenic neurons, and human brain. Together with other readouts and assays, this will help to identify and characterize mitophagy changes in neurodegeneration and in other age-related diseases. The levels of PINK1 protein might serve as a crucial disease or pharmacodynamics marker to analyze the effects of disease modifying therapies that are being developed to overcome PD.

## Results

### Testing commercially available PINK1 antibodies using overexpressed PINK1

**Figure 1.**
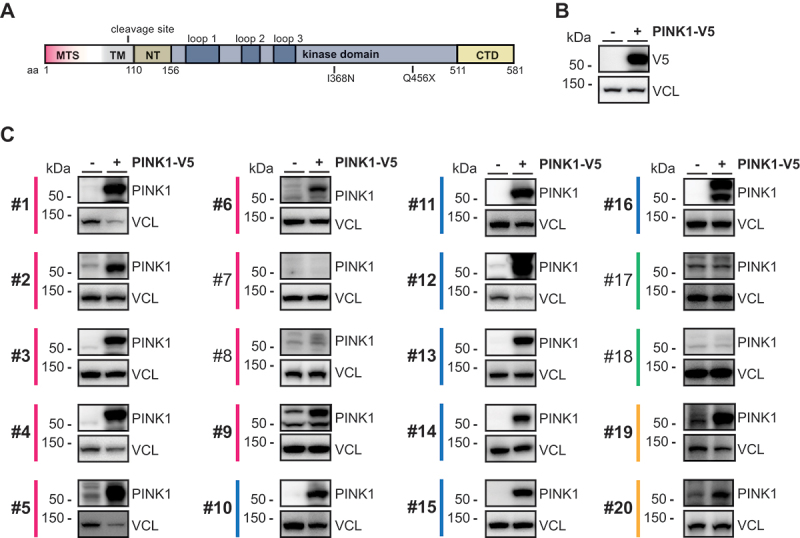
Pre-screening of primary PINK1 antibodies. (A) schematic representation of full-length PINK1 protein, indicating the domain structure, the cleavage site, as well as the positions of the I368N and Q456X mutations. (B, C) *PINK1* knockout (KO) HEK293E cells were transfected either with empty vector (-) or with V5-tagged PINK1 (+) and treated with 20 µM CCCP for 8 h. Western blots were prepared and probed with V5 antibody (B) to control for successful transfection or either of 20 primary PINK1 antibodies (C) to determine their specificity. All primary PINK1 antibodies were used in the identical concentration and are numbered and color-coded by species as in Table 1. Antibodies that were selected for further testing are shown in bold. VCL (vinculin) was used as a loading control.

In order to develop a robust and sensitive PINK1 ELISA, we first prioritized commercially available PINK1 antibodies with either a great number of citations or antibodies that seemed promising with regard to the availability of validation data per the supplier’s website and/or according to recent publications. In total, 20 antibodies directed against the PINK1 protein (Figure 1A) were selected for testing and were derived from either of four different species: rabbit, mouse, goat, and sheep (Table 1). We first evaluated the different PINK1 antibodies in an overexpression paradigm and transfected V5-tagged PINK1 or empty vector into HEK293E *PINK1* knockout (KO) cells. We further treated the transfected cells with the mitochondrial depolarizer carbonyl cyanide 3-chlorophenylhydrazone (CCCP) to stabilize PINK1 protein. A control western blot with V5 antibody showed a strong signal at the expected size of about 60 kDa (Figure 1B). Out of the 20 PINK1 antibodies, 16 detected a band corresponding to full-length PINK1 (Figure 1C). Some antibodies seemed highly specific and showed no signal in the empty vector transfected sample, while others showed some weaker signal in the lane where the control sample had been loaded. Four antibodies (#7, #8, #17, and #18) did not show a discernible for overexpressed PINK1 and were excluded from further analysis.

**Table 1.** List of used primary PINK1 antibodies.

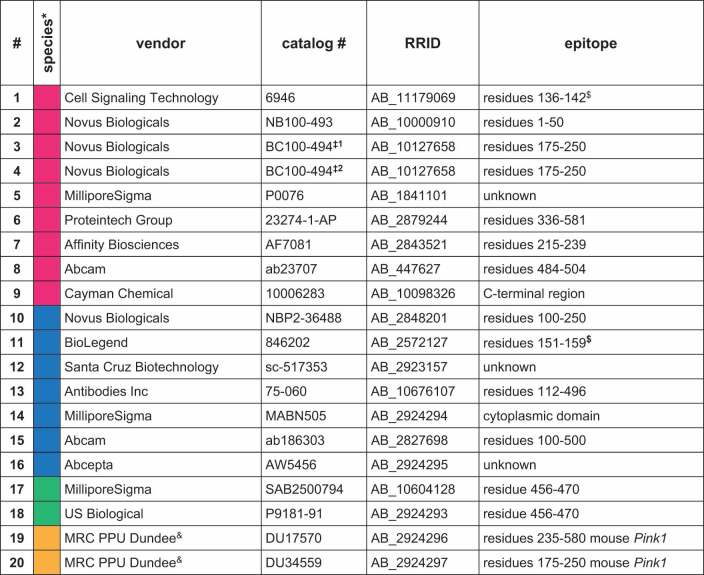

*The host species for each antibody is shown color-coded, pink: rabbit, blue: mouse, green: goat, yellow: sheep. ^$^These epitopes have been determined experimentally (this study). Epitope information for other antibodies is based on the vendor antibody datasheet or website. ^**‡**^Two different lots were tested for this highly cited polyclonal PINK1 antibody: ^1^lot K, ^2^ lot O-2. ^&^MRC Protein Phosphorylation and Ubiquitylation Unit Reagents and Services, University of Dundee.

### Testing of antibodies using samples with endogenous PINK1 levels

**Figure 2.**
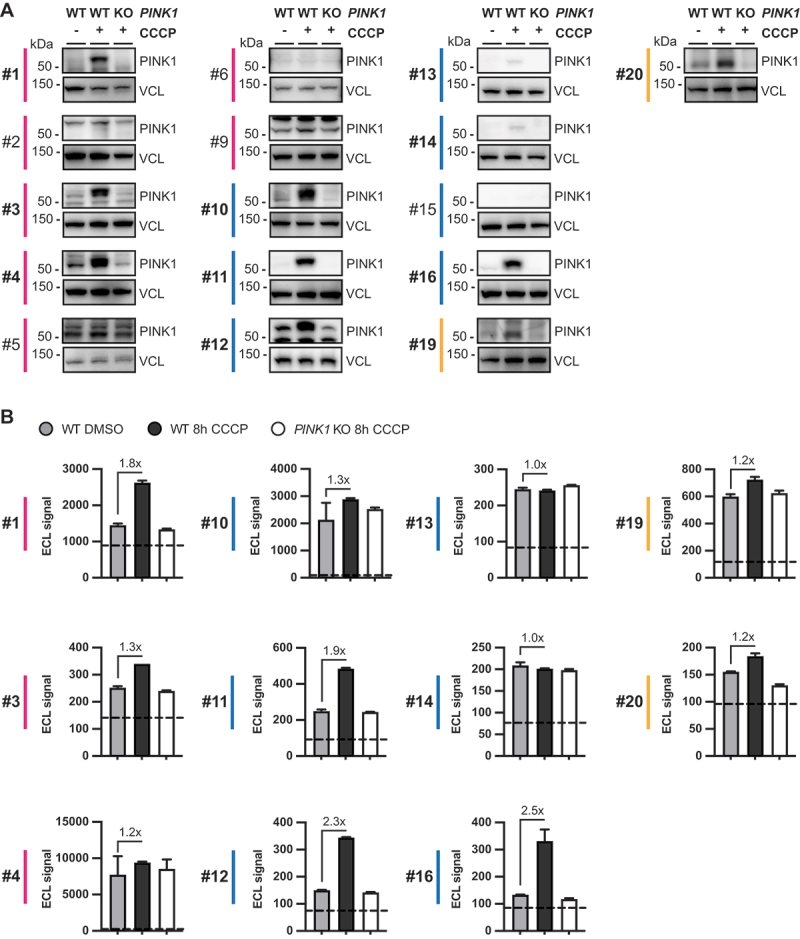
Screening of antibodies to detect endogenous PINK1 protein. Wild-type (WT) or *PINK1* KO HEK293E cells were treated for 8 h with 20 µM CCCP (+). Some cells were treated with vehicle (DMSO) only (-). (A) Western blots were prepared and probed with primary PINK1 antibodies that successfully detected overexpressed PINK1 by western blot (see Figure 1). VCL was used as a loading control. (B) The same RIPA samples were used for a direct ELISA. MSD plates were coated with non-boiled lysates, and primary PINK1 antibodies were used as detection antibodies followed by sulfo-tag labelled secondary antibodies. Samples were measured in duplicates. Shown is the raw ECL signal ± SD. The dashed line indicates the background signal that was obtained when the wells were coated without lysate (buffer control). For each antibody, the fold change of CCCP-treated to DMSO-treated WT samples is indicated on top. Primary PINK1 antibodies are numbered and color-coded by species according to Table 1 and shown in bold if used for further testing.

We next tested the remaining 16 antibodies under endogenous conditions. For this we used lysates from CCCP-treated PINK1 WT HEK293E cells as positive and CCCP-treated *PINK1* KO cells as negative control. We also included samples from WT cells that had only been treated with vehicle (DMSO). Consequently, these samples have very low PINK1 protein levels. Eleven antibodies recognized a band in the treated WT, but no or much weaker signal in the untreated WT or the treated *PINK1* KO samples (Figure 2A). Antibodies #2, #5, #6, #9 and #15 gave either no signal at all, or did not clearly discriminate between positive and negative samples and were excluded from further analysis.

We then tested the remaining eleven antibodies in a direct ELISA on the MSD platform that uses electrochemiluminescence (ECL) as a readout and, compared to traditional colorimetric ELISA, has a better precision and sensitivity due to its large dynamic range. Non-boiled RIPA lysates were used to coat MSD ELISA plates and the different PINK1 antibodies were added as detection antibodies followed by sulfo-tag labeled secondary antibodies (Figure 2B). For each antibody, we also included a blank well that was coated only with the blocking buffer. Only two antibodies did not discriminate between CCCP- and vehicle-treated WT cell samples in this direct ELISA. Four antibodies (#1, #11, #12 and #16) performed well and showed a fold change of over 1.5 between lysates of CCCP-treated to vehicle-treated HEK293E cells. Samples from vehicle-treated WT cells and CCCP-treated *PINK1* KO have very low or no PINK1, respectively, and therefore gave similar results with most antibodies.

### Testing antibody pairs in a sandwich ELISA format

**Figure 3.**
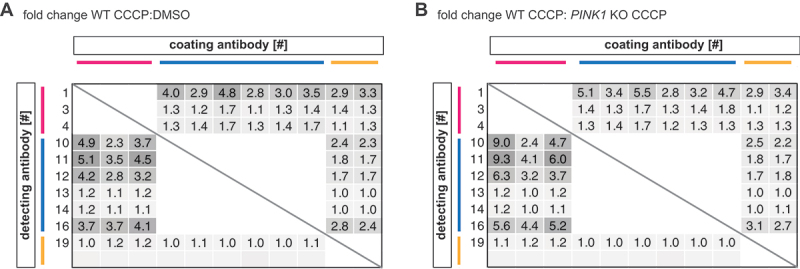
MSD sandwich ELISA antibody pairs. Pairs of PINK1 antibodies were tested in an MSD sandwich ELISA format using samples from WT or *PINK1* KO HEK293E cells were treated for 8 h with 20 µM CCCP (+). Some cells were treated with vehicle (DMSO) only (-). Each antibody pair was used in both directions with either antibody as coating or as detecting antibody, respectively. Shown is a heatmap of (A) the fold change between CCCP- and DMSO-treated *PINK1* WT RIPA lysates and (B) of CCCP-treated WT versus *PINK1* KO RIPA lysates for each antibody combination tested. Darker color indicates a higher fold change. For raw data see Table S1. Same species antibody pairs were not tested. Primary PINK1 antibodies are numbered and color-coded by species according to Table 1.

To potentially increase specificity and sensitivity, we next explored the remaining 11 PINK1 antibodies in all possible combinations in a sandwich ELISA format. Antibody pairs were tested in both orientations with each antibody being used as capture and as detection reagent, respectively. Since the detection of signal relied on sulfo-tag labeled secondary antibodies, pairs of antibodies from the same species were omitted. We used the same sample set as in the previous experiments for all 72 remaining antibody combinations. A table with the raw data can be found as **Table S1**. We calculated the fold change between CCCP- and vehicle-treated WT cells (Figure 3A) as well as CCCP-treated WT and *PINK1* KO cells (Figure 3B) and observed two clusters of promising antibody combinations in the generated heatmaps. Fold changes of 2.9 to 4.8 for CCCP to DMSO treated WT cells, and 2.8 to 5.5 for WT to *PINK1* KO cells, respectively, were achieved when any of the mouse or sheep antibodies was used as coating antibody and the detection was performed with rabbit antibody #1. In contrast, rabbit antibodies #3 and #4 did not work well as detecting antibody.

Similar or even higher fold changes were reached in a second cluster when the wells were coated with any of the three rabbit antibodies and detection occurred with four out of six mouse antibodies (#10, #11, #12, #16). This resulted in fold changes of 2.3 to 5.1 for CCCP to DMSO treated WT cells and 2.4 to 9.3 for WT to *PINK1* KO cells, depending on the exact combination (Figure 3A, B). While coating with the sheep antibodies and detecting with mouse antibodies #10 and #16 also resulted in discrimination between samples with a fold change between 2.2 and 3.1, several rabbit-mouse antibody pairs showed a better discrimination, and we decided to focus on those. Out of the three rabbit antibodies, antibody #1 was superior and we therefore decided to go forward with antibody #1 as capture antibody.

### Comparison of top PINK1 antibody pairs

**Figure 4.**
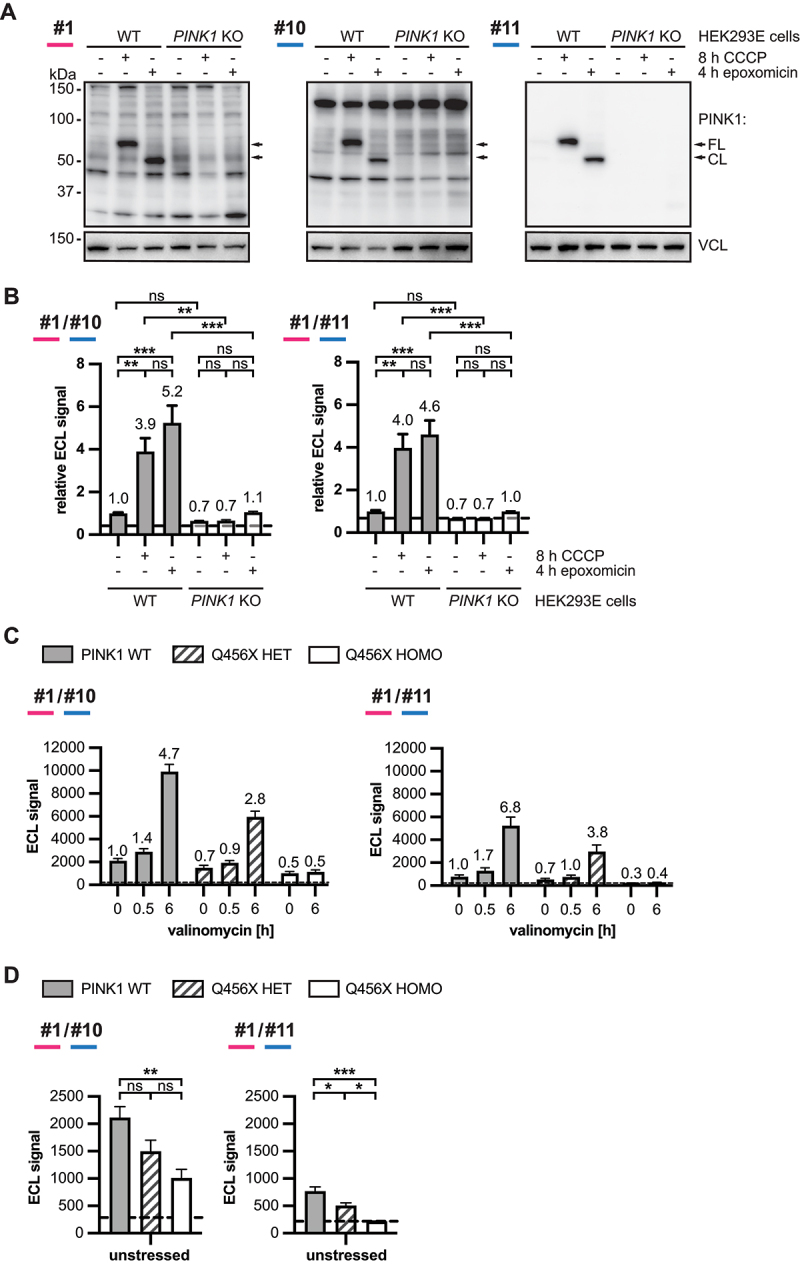
Evaluation of the top performing PINK1 antibodies. (A, B) WT or *PINK1* KO HEK293E cells were treated for 8 h with 20 µM CCCP, for 4 h with 0.1 µM epoxomicin or with DMSO as vehicle (-). (A) Western blots were prepared to test if the best antibodies from the sandwich ELISA test would recognize both the full-length (FL) and the cleaved (CL) form of PINK. VCL was used as loading control. (B) Sandwich ELISA with RIPA samples was performed using antibody #1 as capture antibody paired with either antibody #10 or #11 as detecting antibody, as indicated. Data were normalized to the average of the vehicle-treated WT cells from three independent experiments. Data were analyzed with two-way ANOVA followed by Tukey’s posthoc test for multiple comparisons. (C, D) Fibroblasts without PINK1 mutation (WT) or with hetero- or homozygous PINK1^Q456X^ mutation were either treated with vehicle or were treated with 1 µM valinomycin for 0.5 and/or 6 h. Sandwich ELISA was performed with RIPA lysates using antibody #1 as capture antibody paired with either antibody #10 or #11 as detecting antibody, as indicated. (C) Shown is the average from four independent experiments over time ± SEM. Numbers on top of bars indicate fold changes relative to vehicle treated WT cells for each antibody set. Two-way ANOVA with Tukey post-hoc analysis revealed a significant difference between all three genotypes (not indicated in figure). (D) Data from vehicle treated samples was plotted separately and analyzed with one-way ANOVA followed by Tukey’s posthoc test. Measurements with antibody pair #1/#11 resulted in significant differences between all three genotypes at baseline, in the absence of stress. The dashed lines indicate the background signal that was obtained when the wells were coated without lysate (buffer control).

In order to determine the best performing antibody combinations, we performed additional experiments side-by-side. First, we used HEK293E WT and *PINK1* KO cells that were treated either with vehicle, with CCCP to stabilize full-length PINK1 or with epoxomicin to stabilize the cleaved form of PINK1. Both mouse antibodies #10 and #11 as well as rabbit antibody #1 recognized both the full-length as well as the cleaved form of PINK1 (Figure 4A). Antibody #11 showed fewer unspecific signal on western blots and basically only detected bands corresponding to PINK1 protein, while antibody #10 and rabbit antibody #1 both detected additional bands. Similar results were obtained when using antibody #1 in combination with either #10 or #11 in a sandwich ELISA using the same samples. Either antibody pair showed a robust increase of ECL signal between vehicle- and CCCP-treated WT cells and between the vehicle- and epoxomicin-treated WT cells with similar significance levels (Figure 4B). However, the combination of antibody #1 with antibody #11 resulted in higher significance between CCCP treated WT and *PINK1* KO cells, compared to the #1/#10 antibody pair.

As additional samples, we next used skin fibroblasts carrying either zero, one, or two PINK1^Q456X^ mutant alleles. This mutation is known to cause the loss of PINK1 protein due to nonsense-mediated mRNA decay [27,33]. Sandwich ELISA with either of the two mouse antibodies as detecting reagent resulted in increased ECL signal for WT cells upon 0.5 h and a further increase upon 6 h of valinomycin treatment (Figure 4C). The ELISA with antibody #10 resulted in overall higher ECL signal compared to antibody #11. However, the fold change between stressed and unstressed cells was higher with antibody #11 compared to antibody #10. At 0.5 h the fold change was 1.4 for antibody #10 and 1.7 for antibody #11. At 6 h this fold change increased to 4.7 for antibody #10 and 6.8 for antibody #11. Antibody #10 also showed lower fold changes than antibody #11 with lysates from heterozygous PINK1^Q456X^ cells, which resulted in an about 2-fold lower ECL signal compared to WT cells. In untreated fibroblasts, only antibody #11 resulted in a significant difference between WT and heterozygous Q456X cells as well as between hetero- and homozygous Q456X carrier (Figure 4D). Because of this, we focused on the further characterization of antibody #1 with antibody #11 as the final antibody pair for PINK1 MSD ELISA.

### Detailed characterization of the final PINK1 antibody pair

**Figure 5.**
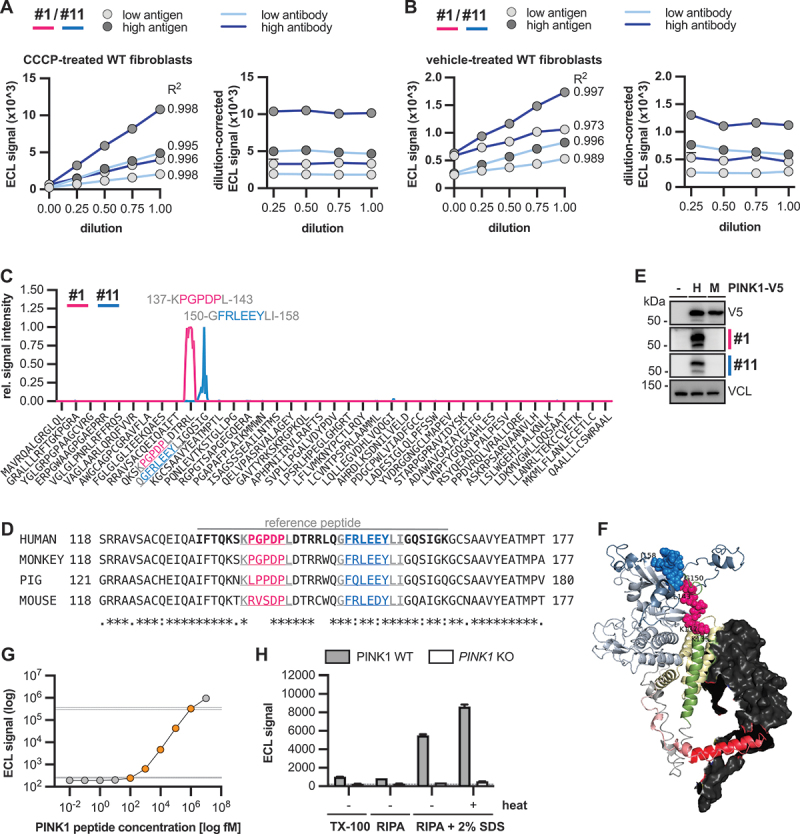
Detailed characterization of the final PINK1 antibody pair. (A, B) Linearity was tested by diluting RIPA lysates from *PINK1* WT fibroblasts, with RIPA lysates from homozygous PINK1^Q456X^ cells in different ratios. Samples were subjected to PINK1 MSD sandwich ELISA with antibody #1 as capture and antibody #11 as detecting antibody. Samples were run in duplicates and different amounts of antigen (low −5 µg, high −15 µg) and coating and detecting antibody (low 1 µg/ml, high −5 µg/ml) were used. We used samples with high PINK1 protein levels from cells that were treated with valinomycin for 6 h (A) or samples with low PINK1 levels from cells that were only treated with vehicle (B). Raw ECL data was plotted (left panels). ECL levels at each dilution were blank subtracted and then multiplied by their respective dilution factor and graphed (right panel). The relatively flat line for each sample type confirms that there is good linearity for this assay in (A) the presence or (B) the absence of induced PINK1. Because the assay is linear for high and low PINK1 concentrations, we can further conclude that there is good parallelism of the assay. (C) Epitope mapping of antibody #1 and #11. Shown is the fluorescence intensity when either antibody was incubated on a peptide array of 15-mer linear PINK1 peptides, followed by incubation with a fluorescence-tagged secondary antibody. Data were averaged and normalized to the highest peak intensity for each antibody. Highlighted are the identified epitopes for antibody #1 (pink color) and #11 (blue color). Residues with uncertainty are shown in gray font. (D) Shown is an alignment of the human PINK1 protein sequence containing both identified epitopes with the corresponding sequence from monkey (*Macaca mulatta*), pig (*Sus scrofa*) and mouse (*Mus musculus*). Highlighted are the epitopes of antibody #1 (pink color) and antibody #11 (blue color). Epitope residues with uncertainty are shown in gray. The peptide that was used as reference peptide to perform absolute quantification is highlighted. (E) *PINK1* knockout (KO) HEK293E cells were transfected either with empty vector (-) or with human (H) or mouse (M) V5-tagged PINK1 and treated with 20 µM CCCP for 8 h. Western blots were prepared and probed with V5 antibody to control for successful transfection as well as PINK1 antibody #1 and #11 to test if they recognize mouse PINK1. VCL was used as loading control. (F) AlphaFold model of human PINK1 with TOMM20. PINK1 is color-coded according to the sequence, while TOMM20 is illustrated as a dark gray surface. The epitope of antibody #1 (K137~L143) and antibody #11 (G150~I158) are shown as pink and blue spheres, respectively. The model is adapted and recolored from [34]. (G) Standard curve using dilutions of reference PINK1 peptide. Shown is the average of three independent measurements of a 10-point standard curve. These data were used to determine the LLOQ and ULOQ which were defined as separated to neighboring concentrations by at least 3 times the SD (dashed lines). The quantitative range of the assay between the LLOQ and ULOQ is indicated in orange. An asymmetric sigmoidal curve fit was used to estimate absolute concentrations of PINK1 in CCCP-treated WT HEK293E cells. (H) WT or *PINK1* KO HEK293E cells were treated for 6 h with 20 µM CCCP and harvested either in buffer containing 1% Triton X-100 or in RIPA. Some aliquots of the RIPA lysates were further denatured by adding 2% SDS either without or with boiling at 95°C for 5 min. Shown is the average of the raw data from the sandwich ELISA with three repeat measurements ± SD. The dashed lines indicate the background signal that was obtained when the wells were coated without lysate (buffer control).

In the absence of available human full-length recombinant PINK1 protein, we tested the linearity of the assay with antibodies #1/#11 in cell lysates. We used lysates from PINK1 WT fibroblasts that had been treated for 6 h with valinomycin and mixed them in defined ratios with lysates from PD patient cells with homozygous PINK1^Q456X^ that do not stabilize PINK1 to generate linear dilutions of PINK1 protein. For the assay, we used either a concentration of 1 or 5 µg/ml for the coating and detecting antibodies and either 5 or 15 µg total cell lysate for the sandwich ELISA, respectively. All four conditions showed a linear dose-dependent increase with R^2^ >0.99. Excellent linearity was also indicated optically when ECL values were plotted after multiplication with the dilution factors (Figure 5A). We next used lysates with lower levels of PINK1 that were generated from unstressed i.e. only vehicle-treated cells. Under this condition, PINK1 protein typically can be hardly detected by western blot, but excellent linearity (R^2^ >0.99) was still obtained when the higher antigen concentration was used (Figure 5B). Because the PINK1 MSD assay showed excellent linearity in samples with high and low PINK1 levels, we can also conclude very good parallelism of the assay [35].

In order to characterize the ELISA assay further, we next mapped the epitopes of both antibodies using peptide microarrays (Figure 5C). For antibody #1, we observed a very strong monoclonal antibody response against a single epitope-like spot pattern formed by adjacent peptides with the consensus motif KPGPDPL (residues 137–143). This result is consistent with the epitope region listed by the manufacturer. For antibody #11, we observed a moderate to strong monoclonal antibody response against adjacent peptides with the consensus motif GFRLEEYLI (residues 150–158), located at the end of the NT domain into the kinase domain and only in short distance to the epitope of antibody #1. While there was perfect homology of both epitopes with rhesus monkey, both epitopes were divergent in mouse (Figure 5D). Experimental testing of transfected V5-tagged mouse PINK1 confirmed that neither antibody recognized mouse PINK1 protein (Figure 5E) although it was expressed at a similar level compared to human PINK1. Given the close distance of both epitopes, we wondered about steric hindrance and used alphafold to visualize the position of the epitopes with in the PINK1 structure [34] (Figure 5F). Both epitopes are located along an outer edge at the end of the NT domain, nestled in between the insertion loops of the kinase domain. The antibodies might be able to bind simultaneously in perpendicular orientation: one upwards, the other sideways. The close distance and quasi-linear orientation of the two epitopes further prompted us try a synthetic peptide as antigen for the assay. A 34-amino acid linear peptide, that contained six additional amino acids on either side (indicated in Figure 5D) was strongly detected by the antibody pair and we use this to generate a 10-point standard curve (R^2^ = 0.9995). The lower limit of quantification (LLOQ) was estimated to be at 100 fM while the upper limit of quantification (ULOQ) was around 1 nM with this peptide (Figure 5G). Side-by-side we measured CCCP-treated PINK1 WT and KO HEK293E cells that had been lysed with either Triton X-100 or RIPA buffer. To aliquots of the RIPA lysates, we further added 2% SDS in some instances and incubated these sample without or with heating at 95°C. While the assay was able to detect PINK1 under all conditions, samples that were boiled in 2% SDS gave by far the highest signal. Since these should be most similar to the linear peptide we used as reference standard, we determined the absolute quantification of PINK1 in these samples. Interpolation of the peptide-derived standard curve revealed an estimated concentration of around 2.3 ng PINK1 protein per mg of CCCP-treated WT HEK293E cell lysate.

### PINK1 measurements in human neurons

**Figure 6.**
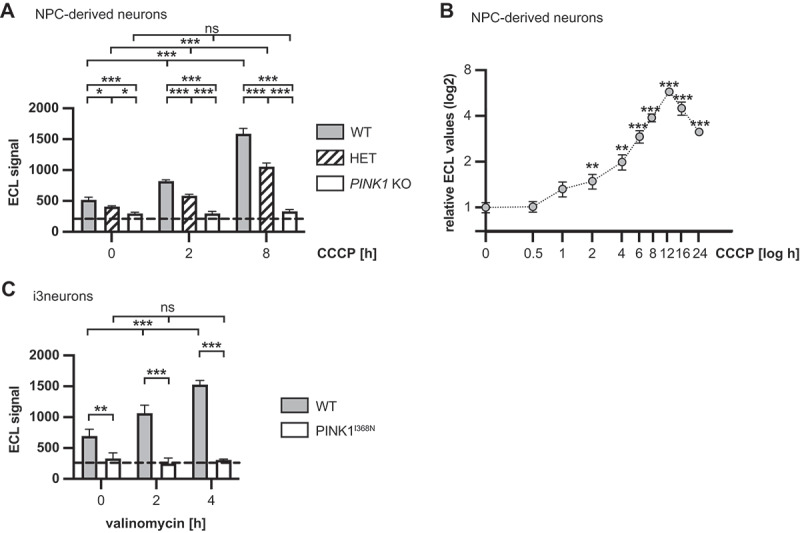
PINK1 levels in neurons. (A) Differentiated isogenic neurons with *PINK1* WT, heterozygous or homozygous *PINK1* KO were treated with 20 µM CCCP for 2 h or 8 h or treated with vehicle (0 h). (B) *PINK1* WT cells were treated with CCCP for the indicated time course. (C) i3neurons generated from iPS cells with homozygous PINK1^I368N^ mutation or isogenic gene-corrected WT cells were treated with 1 µM valinomycin for 2 h or 4 h or treated with vehicle (0 h). Data from three independent experiments is shown for each and graphed as mean -/+ SD. Statistical analysis in A, C was performed with two-way ANOVA followed by Tukey’s posthoc test, in B with unpaired, student’s t-test.

In order to further test the assay with the final antibody pair in disease-relevant cells, we differentiated neurons from precursors in which we had knocked out one or both alleles of *PINK1*. We have previously shown the PINK1-dose dependent generation of p-S65-Ub of these neurons [36]. Consistently, the PINK1 MSD sandwich ELISA with antibodies #1 and #11 was able to distinguish stress- and gene dose-dependent PINK1 protein changes in these cells upon treatment (Figure 6A). WT neurons showed a strong and robust ECL signal increase upon CCCP treatment. In lysates from heterozygous *PINK1* KO neurons, the levels were lower but still significantly increased with CCCP treatment, while the ECL signal did not change upon treatment of homozygous *PINK1* KO neurons. In unstressed neurons, there was a significant difference between all three genotypes too highlighting the sensitivity of the assay. We also explored PINK1 stabilization in WT cells over time using a time course experiment. MSD ECL levels were unchanged after 30 min treatment with CCCP but began to visibly rise after 1 h CCCP treatment (Figure 6B). However, there was some variability between the replicate experiments. A significant difference became apparent after 2-h CCCP treatment. PINK1 levels peaked at 12 h after CCCP after which they declined. It is possible that at time point, PINK1 is starting to be co-degraded together with the damaged mitochondria. To further measure PINK1 in patient-derived neurons, we used induced pluripotent stem cells (iPSC) from a patient with the PINK1^I368N^ mutation. The PINK1^I368N^ mutation is known to cause protein instability, and this has been shown in different cell types, including fibroblasts, undifferentiated iPSCs, and iPSC-derived dopaminergic neurons by western blot [26,36]. Here we generated NEUROG2/NGN2-inducible iPSC that we differentiated into i3neurons [37]. As control, we used an isogenic gene-corrected WT line. The PINK1 MSD sandwich ELISA with antibody pair #1/#11 showed a significant ECL increase in gene-corrected WT cells over time treatment, while the ECL signal from cells with PINK1 remained stable upon CCCP treatment. There was already a significant difference in ECL levels between both genotypes in untreated i3neurons (Figure 6C), further providing evidence that the PINK1 MSD sandwich ELISA sensitively detects PINK1 levels also at baseline in neurons.

### PINK1 detection in human brain

**Figure 7.**
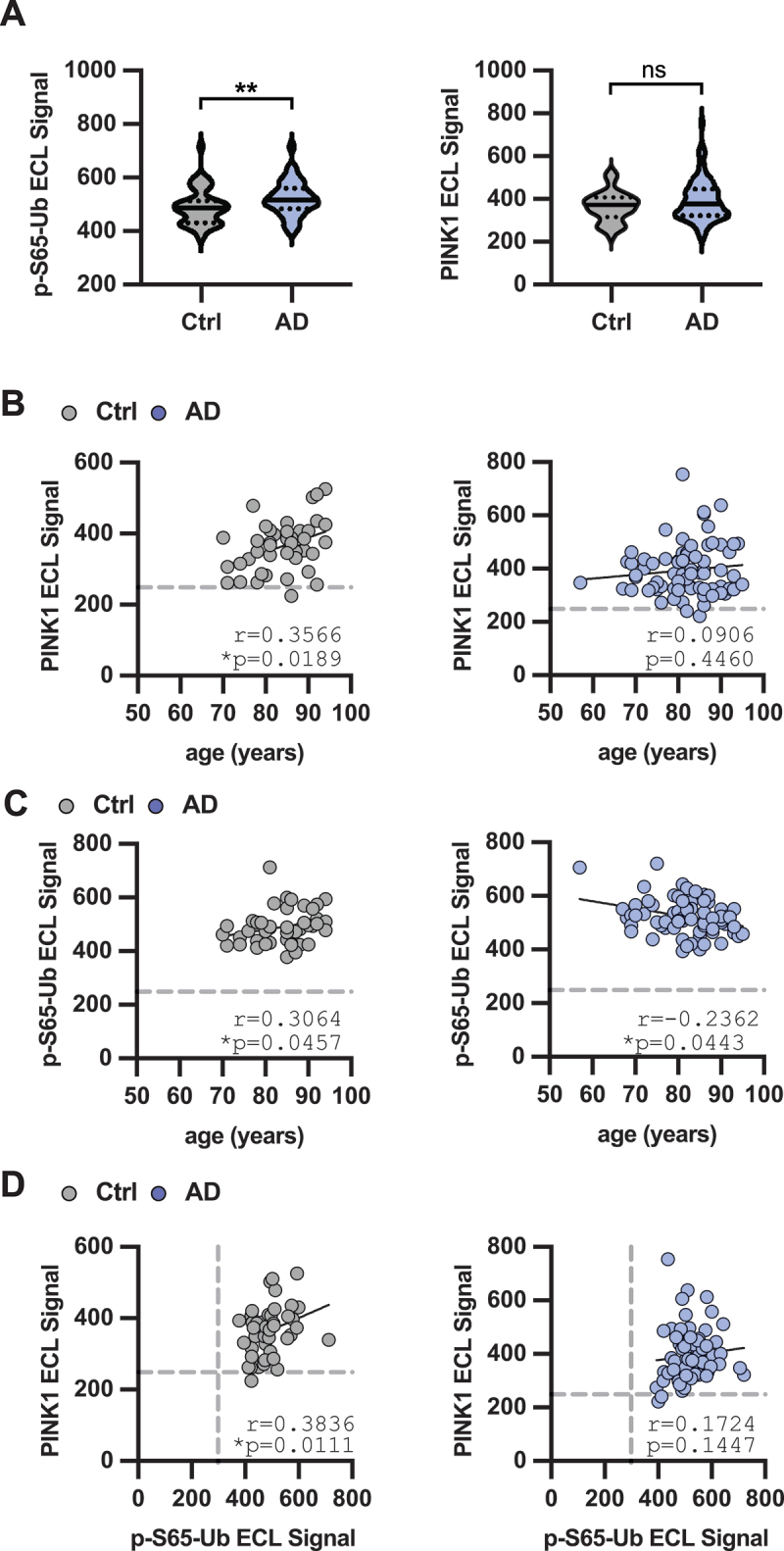
PINK1 measurements in human brain tissue. Frozen midfrontal tissue from neurological normal (controls, n = 43) and AD patients (n = 73) was homogenized, and lysates prepared. Sandwich ELISA for p-S65-Ub and for PINK1 was performed. For PINK1 ELISA antibody #1 was used as capture antibody and antibody #11 as detecting antibody. (A) Violin plot of raw data. A mann-whitney test was used for statistical analysis between groups. Spearman correlation of (B) PINK1 MSD signal or (C) p-S65-Ub MSD signal with age and (D) PINK1 MSD with p-S65-Ub MSD signal was performed separately for each group. Dashed line indicates the value(s) of the buffer blank(s) for each assay. The Spearman correlation coefficient (R) and *p* value are indicated.

We have previously shown that p-S65-Ub levels increase in human brain with age and independently with disease [28,30,31]. In order to establish whether this might be related to an increase in PINK1 protein levels, we used samples from neurologically normal individuals (hereafter referred to as controls) and from individuals with pathologically confirmed Alzheimer disease (AD) (see Table 2 for subject characteristics). We extracted proteins from frozen midfrontal cortex and measured PINK1 and p-S65-Ub by MSD sandwich ELISA. Consistent with our previous findings, p-S65-Ub was significantly increased in AD (Figure 7A). The PINK1 MSD signal showed a larger spread in AD versus controls, but there was no significant difference between controls and AD. Within each group, we performed correlations with age. We found that PINK1 MSD levels significantly correlated with age in controls (*p* = 0.018), but not in the AD group (*p* = 0.43) (Figure 7B). Like PINK1, p-S65-Ub signal also positively correlated with age in controls (*p* = 0.046). However, in the AD group, p-S65-Ub signal was negatively correlated with age (*p* = 0.04) (Figure 7C). There was a positive association of PINK1 with p-S65-Ub in controls (*p* = 0.011) but not AD (*p* = 0.14) (Figure 7D). Collectively these data shows that while p-S65-Ub is increased in AD, this seems to be independent of PINK1 protein levels. There is a positive association of both, PINK1 levels and p-S65-Ub signal, with age in controls

**Table 2.** Subject characteristics.

group	N	N Male (%)	age median (min-max)	Braak tangle stage median (min-max)	Thal amyloid phase median (min-max)
control	43	23 (52)	85 (70–94)	III (0-III)	1 (0–3)
AD	73	38 (52)	82 (57–95)	V (III-VI)	5 (3–5)

Neuropathological scores are presented as median (min-max).

## Discussion

Here we developed a new ELISA method for the detection of the human PINK1 kinase. We screened 20 commercially available PINK1 antibodies first individually and then in combinations in a sandwich ELISA format, where the antigen is bound between a capture and a detection antibody. We have built this assay on the MSD platform that uses electrochemiluminescence as readout and has a larger dynamic range compared to regular colorimetric ELISA for improved sensitivity and precision. We found several antibody pairs that were able to detect PINK1 protein that stabilized upon mitochondrial damage of cells. The best antibody pair consisted of two monoclonal antibodies that bind PINK1 in short distance of each other in the NT region toward the kinase domain. These antibodies resulted in excellent linearity with an estimated the assay range between 100 fM and 1 nM and sufficiently sensitive to detect a significant difference between WT and PINK1 mutant fibroblasts and neurons even at baseline, i.e. in the absence of stress. Under these conditions PINK1 is typically not at all or only faintly detectable by western blot. Thus, this new MSD ELISA offers a superior sensitivity compared to previous methods and will be highly useful to determine the protein levels of PINK1, even under physiological conditions.

In treated HEK293 cells, the MSD ELISA was able to detect both CCCP-induced full-length and epoxomicin-stabilized cleaved form of PINK1 with similar affinity. The ability to bind either of those forms is also consistent with the determined epitopes and it remains unclear if the PINK1 signal that the assay detects at baseline corresponds to full-length or truncated PINK1. Given that PINK1 mutant cells and animals have lower p-S65-Ub levels at baseline [29,36] it seems that at least some of the signal would correspond to active, mitochondrial PINK1. In line with this, the cleaved PINK1 is known to have a very short half-life [8]. However, the ratio of truncated to full-length PINK1 likely varies dependent on many factors such as inherent cellular stress levels or there might be species and tissue specific differences [38]. It should also be noted that truncated PINK1 was suggested to play an important role for neuronal development, plasticity and survival, but this non-mitochondrial role of PINK1 remains not well understood [39].

The activation of the PINK1 kinase is a complex process. Upon damage, when mitochondrial import collapses, PINK1 inserts into the outer mitochondrial membrane prior to dimerization, and autophosphorylation [13,40]. The stabilization of PINK1 at the outer mitochondrial membrane seems dependent on specific intramolecular interaction of the N-terminal with a C-terminal element of PINK1 that are needed for efficient interaction/insertion via the TOMM complex [13,41]. In line with this, several PD-associated mutations located in these domains seem to have reduced PINK1 levels upon CCCP treatment [13,41–43]. It will be interesting to revisit these and other PD-associated PINK1 mutants for effects on PINK1 protein levels at steady state and upon CCCP treatment or other stressors, especially those variants for which a pathogenicity has not been clearly established yet. In line with this, the here developed assay could be very useful to study heterozygous PINK1 mutation carriers with respect to possible differences on the protein level. It should be pointed out though that rare genetic variants that are located within either epitope could affect antibody binding and might not be detected by this assay. Nevertheless, it is still debated whether heterozygous PINK1 (or PRKN) mutation increase the risk for PD and a sensitive method to measure PINK1 levels might help to further address it. While it seems clear that carriers of mutations show dopamine deficits that might contribute to preclinical signs of PD [44–47], large scale genetic analyses do not seem to find clear evidence for increased disease risk [48–51]. It is plausible that the individual risk depends on the expression ratio of WT vs mutant PINK1 allele and therefore the protein levels of active PINK1 kinase. We have previously demonstrated a measurable decline in basal and stress-induced p-S65-Ub levels in the context of heterozygous human patient cells and in mouse brain *in vivo* [36,52]. Interestingly, it has been suggested that the MAPT H1 haplotype confers increased risk for PD by affecting mitophagy through decreasing the protein levels of PINK1 via the NSL complex [53] and the new PINK1 MSD ELISA might be helpful to further investigate this.

Our findings of increased p-S65-Ub levels with age and disease [29–31] suggest a broader role of the mitophagy pathway, but the molecular mechanisms remain unclear. Here, we have used the new MSD ELISA assay to measure PINK1 protein levels in brain samples from neurologically normal individuals or AD patients. In controls, PINK1 levels correlated with age, potentially reflecting the age-dependent increase in mitochondrial stress which might lead to increased generation of p-S65-Ub. In AD brain however, PINK1 levels did neither correlate with age nor with p-S65-Ub levels. This could indicate that the mitophagy label might be accumulating due to a reduced flux rather than elevated mitophagy induction upon PINK1 stabilization. This is in line with a shift toward more lysosomal p-S65-Ub co-localization observed in brain regions with AD pathology [31]. Alternatively, these data might indicate that total protein levels of PINK1 in brain do not reflect its catalytic kinase activity. Clearly more work is needed to investigate the molecular mechanisms of mitophagy alterations in AD.

Despite all the strengths and advantages, the here-developed assay has several limitations. First, it recognizes human but not mouse PINK1 and therefore cannot be used to surrogate PINK1-PRKN mitophagy in preclinical mouse models. However, it should be applicable for larger animal models such as non-human primates. Second, the assay measures total levels of PINK1 independent of its activity. While our current understanding is that total and active PINK1 are strongly correlated, this might not always be the case as for example in AD brain. It is also known that certain mutants have no kinase activity but do not affect the protein stabilization of PINK1. Future work therefore should be directed toward generating a modified version of this assay to detect specifically activated, auto-phosphorylated (p-S228-)PINK1. Third, because we could not detect PINK1 in mass spec data from CCCP-treated neurons (data not shown), we have been unable to corroborate the findings of the PINK1 ELISA. Validation against other orthogonal methods therefore remains to be performed. Fourth, the assay works better on denatured/unfolded PINK1 and hence the equally sensitive detection of folded PINK1 or PINK1 bound to the TOMM complex will require further development efforts. And lastly, more sensitive platforms have been developed and it might be possible to further improve the sensitivity and linear range of the PINK1 ELISA assay by switching to a single molecule counting technology. This technology has been suggested to be more resistant to matrix effects of plasma and therefore might lead to superior performance especially in clinical samples [54,55].

These caveats and opportunities to further improve aside, the here developed PINK1 ELISA as it stands is already much more sensitive compared to any previous detection method for PINK1. Therefore, this assay will be highly useful to study the role of PINK1 and mitophagy under physiological and pathological conditions. Further there are possible diagnostic applications with regards to variants of unknown significance and the individual risk of heterozygous mutation carriers. Together with other assays, the PINK1 ELISA will help to further assess mitophagy as a potential disease and therapeutic biomarker.

## Materials and methods

### Cell culture, treatment and lysis

HEK293E (ATCC, CRL-10852; originally marketed by Invitrogen) cells were cultured in DMEM (Thermo Fisher Scientific, 11965118) with 10% fetal bovine serum (FBS; Neuromics, FBS001800112). For overexpression of V5-tagged PINK1, HEK293E *PINK1* KO cells [29] were transfected with Xtremegene 9 (Millipore Sigma, 6365787001) according to the manufacturer’s instructions for 48 h. V5-tagged human PINK1 was described previously [22]. Mouse *Pink1* cDNA (Genscript, OMu22104) was subcloned into the same pcDNA6-V5-6×His backbone using NheI/XbaI restriction sites.

Primary human dermal fibroblasts were collected under an approved Mayo Clinic ethical review board protocol (09–003803). Cells with homo- or heterozygous PINK1^Q456X^ have been described previously [27,52]. Control cells without PINK1 mutation were collected from a related individual. Fibroblasts were maintained in DMEM containing 10% FBS, 0.5% PenStrep (Thermo Fisher Scientific, 15140122) and 1% non-essential amino acids (Thermo Fisher Scientific, 11140050).

Differentiated ReN cell VM (Millipore, SCC008), as well as gene-edited derivatives with hetero- and homozygous *PINK1* KO have been described previously [36,56]. ReN cells were grown on Matrigel matrix (Corning, 354230) in DMEM-F12 media (Thermo Fisher Scientific, 10565042) supplemented with B-27 (Thermo Fisher Scientific, 17504044), 5 U/ml heparin (Sigma Aldrich, H3149), and 50 μg/ml gentamicin (Thermo Fisher Scientific, 15750060). For proliferation, the media was further supplemented with 20 ng/ml FGF (fibroblast growth factor; Peprotech, 100–18B) and EGF (epidermal growth factor; Peprotech, AF-100-15). Differentiation of cells was induced by withdrawing the growth factors in the presence 1 mm dibutyryl-cAMP (Invivochem, V1846) and 2 ng/ml GDNF (glial cell derived neurotrophic factor; Peprotech, 450–10) for at least 15 days. Cells were treated in differentiation medium with B-27 that contained no antioxidants (Thermo Fisher Scientific, 10889038).

Human induced pluripotent stem cells (iPSCs) were received from the NINDS stem cell repository (https://stemcells.nindsgenetics.org, PINK1^I368N^: ND50010, gene-corrected WT PINK1: ND50012). IPSCs were maintained on a Matrigel matrix (Corning, 354277) and fed with mTeSR Plus (Stemcell Technologies, 100–0276) every other day.

All cells were maintained at 37°C and 5% CO_2_ in a humidified atmosphere. Cells were treated with 20 µM CCCP (Sigma Aldrich, C2759), 1 µM valinomycin (Cayman Chemical, 10009152) or 100 nM epoxomicin (Selleck Chemicals, S7038) to induce PINK1 stabilization where indicated. Control cells were treated with DMSO vehicle (Sigma Aldrich, D4540). Cells were harvested in 1× RIPA (50 mM Tris [Thermo Scientific, J75825-A4], pH 8.0, 150 mM NaCl [Fisher Scientific, BP358–10], 0.1% SDS [Fisher Bioreagents, BP166–500], 0.5% deoxycholate [Sigma, D6750], 1% NP-40/Igepal [United States Biochemical, 19628]) with protease and phosphatase inhibitors (Roche Applied Science, 04906845001, 05056489001). For one set of experiments, some cells were lysed a buffer containing 50 mM Tris, pH 8.0, 150 mM NaCl and 1% Triton X-100 (Sigma, X100). Lysates were cleared at 20,000 g for 15 min at 4°C before protein measurements using a bicinchoninic acid assay (ThermoFisher, 23225).

### Gene-editing of iPSCs and differentiation to i3Ns

PINK1^I368N^ and gene-corrected WT cells were gene-edited to insert a doxycycline-inducible NEUROG2/NGN2 cassette (Addgene, 124229; deposited by Michael Ward) into the CLYBL safe harbor locus for the generation of i3neurons (i3Ns) [37]. A detailed protocol is available as accompanying document on the Addgene website. In brief, iPSCs were transiently transfected with a ribonucleoprotein complex of sgRNA (Synthego, custom sequence ATGTTGGAAGGATGAGGAAA) and Cas9 (Integrated DNA Technologies, 1081059) and with 500 ng of donor plasmid as well as 100 ng of a dominant negative TP53/p53 plasmid (Addgene, 41856; deposited by Shinya Yamanaka). Cells were individualized the next day and selected with 50 µg/ml blasticidin (invivogen, ant-bl-1). Individual clones were picked, confirmed by PCR and by successful differentiation to i3N. IPSCs were differentiated into i3Ns using a previously published protocol with only minor modifications [37]. In brief, once the iPSCs reached about 75% confluency, they were dissociated using Accutase (Millipore, SCR005) and seeded at 1 × 10^6^ cells/well onto a Matrigel (Corning, 354277)-coated 6-well plate using KnockOut Dulbecco’s Modified Eagle Medium (DMEM)/F12 (Thermo Fisher Scientific, 12660012) containing 1% *N*-2 supplement (Thermo Fisher Scientific, 17502048), 1% Glutamax (Thermo Fisher Scientific, 35050061), 1% MEM Non-Essential Amino Acids (NEAA) solution (Thermo Fisher Scientific, 11140050), 2 µg/mL doxycycline (Applichem, A2951), and 10 µM Y-27632 ROCK inhibitor (Selleckchem, S1049). Full media changes were subsequently performed for the next 3 days without ROCK inhibitor, and doxycycline was freshly supplemented to the media each day. On day 4, the cells were again dissociated using Accutase and transferred onto a 0.1 mg/ml poly-L-ornithine/PLO (Sigma Aldrich, P3655) and 10 µg/ml laminin (Sigma Aldrich, L2020)-coated 6-well plate at 1 × 10^6^ cells/well using KnockOut DMEM/F12 and Neurobasal A Medium (Thermo Fisher Scientific, 10888022) containing 0.5% *N*2 supplement, 1% B27 supplement (Thermo Fisher Scientific, 17504044), 1% Glutamax, 1% NEAA, 1 µg/ml laminin, 10 ng/mL BDNF (brain derived neurotrophic factor; Peprotech, 450–02), 10 ng/ml NTF3 (neurotrophin 3; Peprotech, 450–03), and 2 µg/ml doxycycline. Half-media changes were subsequently performed every 3 days until harvesting between days 10–14.

### Brain tissue

Brain tissue from 43 neurologically normal controls and 73 subjects with pathologically confirmed Alzheimer disease was obtained from the Mayo Clinic Florida Brain Bank. All brains were examined in a systematic and standardized manner. Neuropathological characteristics of these 2 groups are summarized in Table 2 and include age at death, Braak tangle stage (0-VI) as well as Thal amyloid phase (0–5) [57,58]. The Mayo Clinic Brain operates with approval of the Mayo Clinic Institutional Review Board. All brain samples are from autopsies performed after approval by the legal next-of-kin. Research on de-identified postmortem brain tissue is considered exempt from human subject regulations by the Mayo Clinic Institutional Review Board.

Sections from midfrontal cortex were stored at −80°C and remained frozen on dry ice during weighing. The tissue was homogenized using 2-ml glass homogenizers (Fisher Scientific, K885300–0002) using 5× volumes of ice-cold Tris-buffered saline (TBS) buffer (50 mM Tris, pH 7.4, 150 mM NaCl) containing phosphatase and protease inhibitors. Both plunger A and B, were used for 20 strokes each. Tissue lysis and protein extraction was completed by adding 25 µl of a 5× RIPA buffer (250 mM Tris, pH 8.0, 750 mM NaCl, 0.5% SDS, 2.5% deoxycholate, 5% NP-40/Igepal) to 100 µl TBS homogenate. The samples were subsequently vortexed and incubated for 30 min on ice. After incubation, lysates were transferred to ultracentrifugation tubes (Beckman, 357448) and spun at 100K x g for 30 min at 4°C. The resulting supernatant was transferred to a new tube. Protein concentrations were determined using a bicinchoninic acid assay.

### Western blot

Protein lysates were prepared in Laemmli buffer (62.5 mM Tris pH 6.8, 1.5% SDS, 8.33% glycerol [Fisher Scientific, BP2291], 1.5% β-mercaptoethanol [Sigma Aldrich, M3148], 0.005% bromophenol blue [Sigma Aldrich, B5525]) and boiled at 95°C for five min. The protein electrophoresis was performed using 8–16% Tris-glycine gels (Invitrogen, XP08165BOX) using a standard running buffer (25 mM Tris, pH 8.3, 0.2 M glycine, 0.1% SDS) at room temperature. Twenty µg protein was loaded per well. Proteins were transferred onto polyvinylidene fluoride/PVDF membranes (Millipore, IEVH00005). For antibodies from rabbit or mouse, blocking was performed with 5% nonfat milk (Sysco, 5398953) in TBS containing 1% Tween-20 (Sigma, P1379; TBST). For antibodies from goat or sheep 10% horse serum (Thermo Fisher Scientific, 16050122) in TBST was used. This was followed by incubation with primary PINK1 antibodies at 1 µg/ml concentration in 5% BSA (Boston Bioproducts, *P =* 753) in TBST (for mouse and rabbit antibodies) or 10% horse serum (for goat and sheep antibodies) at 4°C overnight. V5 antibody (Invitrogen, R960, 1:5000) was used to confirm the expression of V5-tagged PINK1. VCL (vinculin; Sigma-Aldrich, V9131) was used as a loading control at a 1:375,000 dilution in 5% milk in TBST for 1 h at room temperature. Next, the membranes were washed for 30 min in TBST then incubated with a secondary HRP-conjugated antibody (Jackson ImmunoResearch, 111-035-003 [anti-rabbit], 715-035-150 [anti-mouse]; Santa Cruz Biotechnology, sc-2020 [anti-goat]; Fisher Scientific, 31480 [anti-sheep]) in 5% milk in TBST for 1 h at room temperature. Signal was developed using an HRP Substrate (Millipore, WBKLS0500) and imaged on a ChemiDoc MP imaging system (Bio-Rad, Hercules, CA, USA).

### Direct ELISA

MSD plates [Meso Scale Diagnostics, L15XA–3, lot: 21L39A] were coated with 5 µg of unboiled HEK293E RIPA cell lysates per well in 30 µl volume. Lysates were diluted with 200 mM sodium carbonate buffer, pH 9.7 and were incubated at 4C overnight in duplicates. The next day, the plates were washed with TBST, and the wells were blocked for 1 h with 300 µl of 1% BSA (for antibodies from mouse and rabbit) or horse serum (for goat and sheep antibodies) in TBST. Next, 30 µl of diluted primary anti-PINK1 antibody (final concentration 1 µg/ml) was added to each well for a 2-h incubation at 600 rpm. The plates were washed with TBST and 50 µl of Sulfo-Tag labeled secondary antibodies (Meso Scale Diagnostics, R32AB, R32AC, R32AI) were added to each well at a 1:500 dilution for 1-h incubation at 600 rpm. After washing, 150 µl of MSD Gold Read Buffer (Meso Scale Diagnostics, R92TG–2) was added to each well and the plates were read on a MESO QuickPlex SQ 120 reader (Meso Scale Discovery, Rockville, MD, USA).

### Sandwich ELISA

For sandwich ELISA, MSD plates were coated with 30 µl of PINK1 capturing in 200 mm sodium carbonate buffer, pH 9.7. Plates were incubated overnight at 4°C, washed with TBST and then blocked either with 300 µl of 1% BSA (for mouse and rabbit antibodies) or 10% horse serum (for goat and sheep antibodies) in TBST for 1 h at room temperature. Next, 30 µl of the cell lysates were added to the plates in duplicates with 5 µg/well for HEK293E and ReN cells, 10 µg/well for patient fibroblasts and human brain samples, and 4 µg/well for i3neurons, unless indicated otherwise. Lysates were diluted in 1% BSA or 10% horse serum in TBST to a final concentration of 10% lysis buffer and were incubated for 2 h at 600 rpm. For one set of experiments 1/5 volume of 10% SDS was added to the RIPA lysates and the samples were then either incubated at room temperature or at 95C for 5 min. These samples were diluted to 0.15% SDS before they were loaded onto the ELISA plate and incubated for 2 h at 600 rpm. The plates were washed and then 30 µl of PINK1 detecting antibodies were added to each well for further incubation of 2 h at 600 rpm. The concentration for the capture and detecting PINK1 antibodies were 1 µg/ml unless indicated otherwise. The plates were washed again and 50 µl of 1:500 (commercial recommendation) Sulfo-Tag labeled antibodies were added to each well for 1-h incubation at 600 rpm. Finally, 150 µl of the MSD Gold Read Buffer was added to each well before reading.

### Epitope mapping, peptide synthesis and standard curves

Epitope mapping was performed by a third party (PEPperPRINT GmbH, Heidelberg, Germany). In brief, the sequence of PINK1 was elongated with neutral GSGSGSG linkers at the C and N termini to avoid truncated peptides and converted into linear 15 amino acid peptides with a peptide-peptide overlap of 14 amino acids. The resulting PINK1 peptide microarrays contained 581 different peptides printed in duplicate and were framed by additional HA (YPYDVPDYAG, 44 spots) and MYC/c-Myc (EQKLISEEDL, 42 spots) control peptides. The microarray was incubated with primary PINK1 antibodies in a concentration of 1 µg/ml in incubation buffer, followed by staining with secondary and control antibodies. Imaging was performed using a LI-COR Odyssey Imaging System at scanning intensities of 7/7 (red/green). The additional HA control peptides framing the peptide microarray were simultaneously stained with the control antibody as internal quality control to confirm the assay quality and the peptide microarray integrity. Spot intensities were analyzed using 16-bit gray scale tiff files and background corrected.

To approximate absolute concentrations of PINK1, we synthesized the following peptide with a free N and acid C terminus at the Mayo Clinic proteomics core: IFTQKSKPGPDPLDTRRLQGFRLEEYLIGQSIGK. The concentration of the generated peptide was determined using absorption at 280 nm prior to lyophilization. The peptide was then solubilized in PBS and serial dilutions for ELISA were prepared in blocking buffer containing 10% RIPA buffer. Three replicate standard curves were used to determining the range of the assay. The LLOQ and ULOQ were defined as concentrations where repeat measurements had an imprecision of less than 5% and were separated at least by three times the standard deviation from the measurements of neighboring standards on both sides [59]. An asymmetric sigmoidal standard curve was interpolated (GraphPad Prism version 10.3.1).

### Statistical analysis

Data was graphed and analyzed with GraphPad Prism (version 10.3.1). Statistical analysis of cell data was performed with one-way or two-way ANOVA followed by Tukey’s t-test as indicated. Time course data of differentiated neurons was analyzed with student’s t-test. Analysis of human brain samples was performed using a non-parametric Mann-Whitney comparisons and spearman correlations. Statistical significance is indicated as follows: (ns, not significant; *, *p* < 0.05; **, *p* < 0.01; ***, *p* < 0.001).

## Supplementary Material

Baninameh_SUPPL_R3.docx

## References

[1] Truban D, Hou X, Caulfield TR, et al. PINK1, Parkin, and mitochondrial quality control: what can we learn about Parkinson’s disease pathobiology? J Parkinsons Dis. 2017;7(1):13–29. doi: 10.3233/JPD-160989 27911343 PMC5302033

[2] Unoki M, Nakamura Y. Growth-suppressive effects of BPOZ and EGR2, two genes involved in the PTEN signaling pathway. Oncogene. 2001 Jul 27;20(33):4457–4465. doi: 10.1038/sj.onc.1204608 11494141

[3] Goncalves FB, Morais VA. PINK1: a bridge between mitochondria and Parkinson’s disease. Life (Basel). 2021 Apr 21;11(5):371. doi: 10.3390/life11050371 33919398 PMC8143285

[4] Vizziello M, Borellini L, Franco G, et al. Disruption of mitochondrial homeostasis: the role of PINK1 in Parkinson’s disease. Cells. 2021 Nov 4;10(11):3022. doi: 10.3390/cells10113022 34831247 PMC8616241

[5] Greene AW, Grenier K, Aguileta MA, et al. Mitochondrial processing peptidase regulates PINK1 processing, import and Parkin recruitment. EMBO Rep. 2012 Apr;13(4):378–385. doi: 10.1038/embor.2012.14 22354088 PMC3321149

[6] Deas E, Plun-Favreau H, Gandhi S, et al. PINK1 cleavage at position A103 by the mitochondrial protease PARL. Hum Mol Genet. 2011 Mar 1;20(5):867–879. doi: 10.1093/hmg/ddq526 21138942 PMC3033179

[7] Jin SM, Lazarou M, Wang C, et al. Mitochondrial membrane potential regulates PINK1 import and proteolytic destabilization by PARL. J Cell Biol. 2010 Nov 29;191(5):933–942. doi: 10.1083/jcb.201008084 21115803 PMC2995166

[8] Yamano K, Youle RJ. PINK1 is degraded through the N-end rule pathway. Autophagy. 2013 Nov 1;9(11):1758–1769. doi: 10.4161/auto.24633 24121706 PMC4028335

[9] Lin W, Kang UJ. Characterization of PINK1 processing, stability, and subcellular localization. J Neurochem. 2008 Jul;106(1):464–474. doi: 10.1111/j.1471-4159.2008.05398.x 18397367 PMC3638740

[10] Sekine S, Wang C, Sideris DP, et al. Reciprocal roles of Tom7 and OMA1 during mitochondrial import and activation of PINK1. Mol Cell. 2019 Mar 7;73(5):1028–1043 e5. doi: 10.1016/j.molcel.2019.01.002 30733118

[11] Okatsu K, Uno M, Koyano F, et al. A dimeric PINK1-containing complex on depolarized mitochondria stimulates Parkin recruitment. J Biol Chem. 2013 Dec 20;288(51):36372–36384. doi: 10.1074/jbc.M113.509653 24189060 PMC3868751

[12] Aerts L, Craessaerts K, De Strooper B, et al. PINK1 kinase catalytic activity is regulated by phosphorylation on serines 228 and 402. J Biol Chem. 2015 Jan 30;290(5):2798–2811. doi: 10.1074/jbc.M114.620906 25527497 PMC4317039

[13] Rasool S, Veyron S, Soya N, et al. Mechanism of PINK1 activation by autophosphorylation and insights into assembly on the TOM complex. Mol Cell. 2022 Jan 6;82(1):44–59 e6. doi: 10.1016/j.molcel.2021.11.012 34875213

[14] Kane LA, Lazarou M, Fogel AI, et al. PINK1 phosphorylates ubiquitin to activate Parkin E3 ubiquitin ligase activity. J Cell Biol. 2014 Apr 28;205(2):143–153. doi: 10.1083/jcb.201402104 24751536 PMC4003245

[15] Kazlauskaite A, Kondapalli C, Gourlay R, et al. Parkin is activated by PINK1-dependent phosphorylation of ubiquitin at Ser65. Biochem J. 2014 May 15;460(1):127–139. doi: 10.1042/BJ20140334 24660806 PMC4000136

[16] Koyano F, Okatsu K, Kosako H, et al. Ubiquitin is phosphorylated by PINK1 to activate parkin. Nature. 2014 Jun 5;510(7503):162–166. doi: 10.1038/nature13392 24784582

[17] Iguchi M, Kujuro Y, Okatsu K, et al. Parkin-catalyzed ubiquitin-ester transfer is triggered by PINK1-dependent phosphorylation. J Biol Chem. 2013 Jul 26;288(30):22019–22032. doi: 10.1074/jbc.M113.467530 23754282 PMC3724655

[18] Kondapalli C, Kazlauskaite A, Zhang N, et al. PINK1 is activated by mitochondrial membrane potential depolarization and stimulates Parkin E3 ligase activity by phosphorylating serine 65. Open Biol. 2012 May;2(5):120080. doi: 10.1098/rsob.120080 22724072 PMC3376738

[19] Shiba-Fukushima K, Imai Y, Yoshida S, et al. PINK1-mediated phosphorylation of the parkin ubiquitin-like domain primes mitochondrial translocation of parkin and regulates mitophagy. Sci Rep. 2012;2(1):1002. doi: 10.1038/srep01002 23256036 PMC3525937

[20] Abou-Sleiman PM, Muqit MM, McDonald NQ, et al. A heterozygous effect for PINK1 mutations in Parkinson’s disease? Ann Neurol. 2006 Oct;60(4):414–419. doi: 10.1002/ana.20960 16969854

[21] Ordureau A, Sarraf SA, Duda DM, et al. Quantitative proteomics reveal a feedforward mechanism for mitochondrial PARKIN translocation and ubiquitin chain synthesis. Mol Cell. 2014 Nov 6;56(3):360–375. doi: 10.1016/j.molcel.2014.09.007 25284222 PMC4254048

[22] Geisler S, Holmstrom KM, Skujat D, et al. PINK1/Parkin-mediated mitophagy is dependent on VDAC1 and p62/SQSTM1. Nat Cell Biol. 2010 Feb;12(2):119–131. doi: 10.1038/ncb2012 20098416

[23] Lazarou M, Sliter DA, Kane LA, et al. The ubiquitin kinase PINK1 recruits autophagy receptors to induce mitophagy. Nature. 2015 Aug 20;524(7565):309–314. doi: 10.1038/nature14893 26266977 PMC5018156

[24] Heo JM, Ordureau A, Paulo JA, et al. The PINK1-PARKIN mitochondrial ubiquitylation pathway drives a program of OPTN/NDP52 recruitment and TBK1 activation to promote Mitophagy. Mol Cell. 2015 Oct 1;60(1):7–20. doi: 10.1016/j.molcel.2015.08.016 26365381 PMC4592482

[25] Fiesel FC, James ED, Hudec R, et al. Mitochondrial targeted HSP90 inhibitor gamitrinib-tpp (G-TPP) induces PINK1/Parkin-dependent mitophagy. Oncotarget. 2017 Dec 5;8(63):106233–106248. doi: 10.18632/oncotarget.22287 29290944 PMC5739729

[26] Ando M, Fiesel FC, Hudec R, et al. The PINK1 p.I368N mutation affects protein stability and ubiquitin kinase activity. Mol Neurodegener. 2017 Apr 24;12(1):32. doi: 10.1186/s13024-017-0174-z 28438176 PMC5404317

[27] Siuda J, Jasinska-Myga B, Boczarska-Jedynak M, et al. Early-onset Parkinson’s disease due to PINK1 p.Q456X mutation – clinical and functional study. Parkinsonism Relat Disord. 2014 Nov;20(11):1274–1278. doi: 10.1016/j.parkreldis.2014.08.019 25226871 PMC4253017

[28] Fiesel FC, Ando M, Hudec R, et al. (Patho-)physiological relevance of PINK1-dependent ubiquitin phosphorylation. EMBO Rep. 2015 Sep;16(9):1114–1130. doi: 10.15252/embr.201540514 26162776 PMC4576981

[29] Watzlawik JO, Hou X, Fricova D, et al. Sensitive elisa-based detection method for the mitophagy marker p-S65-ub in human cells, autopsy brain, and blood samples. Autophagy. 2021 Sep;17(9):2613–2628. doi: 10.1080/15548627.2020.1834712 33112198 PMC8496550

[30] Hou X, Fiesel FC, Truban D, et al. Age- and disease-dependent increase of the mitophagy marker phospho-ubiquitin in normal aging and lewy body disease. Autophagy. 2018;14(8):1404–1418. doi: 10.1080/15548627.2018.1461294 29947276 PMC6372017

[31] Hou X, Watzlawik JO, Cook C, et al. Mitophagy alterations in Alzheimer’s disease are associated with granulovacuolar degeneration and early tau pathology. Alzheimers Dement. 2020 Oct 8;17(3):417–430. doi: 10.1002/alz.12198 33090691 PMC8048674

[32] Shiba-Fukushima K, Ishikawa KI, Inoshita T, et al. Evidence that phosphorylated ubiquitin signaling is involved in the etiology of Parkinson’s disease. Hum Mol Genet. 2017 Aug 15;26(16):3172–3185. doi: 10.1093/hmg/ddx201 28541509

[33] Grunewald A, Gegg ME, Taanman JW, et al. Differential effects of PINK1 nonsense and missense mutations on mitochondrial function and morphology. Exp Neurol. 2009 Sep;219(1):266–273. doi: 10.1016/j.expneurol.2009.05.027 19500570

[34] Raimi OG, Ojha H, Ehses K, et al. Mechanism of human PINK1 activation at the TOM complex in a reconstituted system. Sci Adv. 2024 Jun 7;10(23):eadn7191. doi: 10.1126/sciadv.adn7191 38848361 PMC11160474

[35] Lee JW, Devanarayan V, Barrett YC, et al. Fit-for-purpose method development and validation for successful biomarker measurement. Pharm Res. 2006 Feb;23(2):312–328. doi: 10.1007/s11095-005-9045-3 16397743

[36] Watzlawik JO, Fiesel FC, Fiorino G, et al. Basal activity of PINK1 and PRKN in cell models and rodent brain. Autophagy. 2024 May;20(5):1147–1158. doi: 10.1080/15548627.2023.2286414 38041584 PMC11135862

[37] Fernandopulle MS, Prestil R, Grunseich C, et al. Transcription factor–mediated differentiation of human iPSCs into neurons. Curr Protoc Cell Biol. 2018 Jun;79(1):e51. doi: 10.1002/cpcb.51 29924488 PMC6993937

[38] Yang W, Guo X, Tu Z, et al. PINK1 kinase dysfunction triggers neurodegeneration in the primate brain without impacting mitochondrial homeostasis. Protein Cell. 2022 Jan;13(1):26–46. doi: 10.1007/s13238-021-00888-x 34800266 PMC8776976

[39] Soman SK, Dagda RK. Role of cleaved PINK1 in neuronal development, synaptogenesis, and plasticity: implications for Parkinson’s disease. Front Neurosci. 2021;15:769331. doi: 10.3389/fnins.2021.769331 34795558 PMC8593325

[40] Gan ZY, Callegari S, Cobbold SA, et al. Activation mechanism of PINK1. Nature. 2022 Feb;602(7896):328–335. doi: 10.1038/s41586-021-04340-2 34933320 PMC8828467

[41] Eldeeb MA, Bayne AN, Fallahi A, et al. Tom20 gates PINK1 activity and mediates its tethering of the TOM and TIM23 translocases upon mitochondrial stress. Proc Natl Acad Sci USA. 2024 Mar 5;121(10):e2313540121. doi: 10.1073/pnas.2313540121 38416681 PMC10927582

[42] Broadway BJ, Boneski PK, Bredenberg JM, et al. Systematic functional analysis of PINK1 and PRKN coding variants. Cells. 2022 Aug 5;11(15):2426. doi: 10.3390/cells11152426 35954270 PMC9367835

[43] Ma KY, Fokkens MR, van Laar T, et al. Systematic analysis of PINK1 variants of unknown significance shows intact mitophagy function for most variants. NPJ Parkinsons Dis. 2021 Dec 10;7(1):113. doi: 10.1038/s41531-021-00258-8 34893635 PMC8664852

[44] Pavese N, Khan NL, Scherfler C, et al. Nigrostriatal dysfunction in homozygous and heterozygous parkin gene carriers: an 18F-dopa PET progression study. Mov Disord. 2009 Nov 15;24(15):2260–2266. doi: 10.1002/mds.22817 19845000

[45] Hilker R, Klein C, Ghaemi M, et al. Positron emission tomographic analysis of the nigrostriatal dopaminergic system in familial parkinsonism associated with mutations in the parkin gene. Ann Neurol. 2001 Mar;49(3):367–376. doi: 10.1002/ana.74 11261512

[46] Klein C, Lohmann-Hedrich K, Rogaeva E, et al. Deciphering the role of heterozygous mutations in genes associated with parkinsonism. Lancet Neurol. 2007 Jul;6(7):652–662. doi: 10.1016/S1474-4422(07)70174-6 17582365

[47] Binkofski F, Reetz K, Gaser C, et al. Morphometric fingerprint of asymptomatic Parkin and PINK1 mutation carriers in the basal ganglia. Neurology. 2007 Aug 28;69(9):842–850. doi: 10.1212/01.wnl.0000267844.72421.6c 17724286

[48] Krohn L, Grenn FP, Makarious MB, et al. Comprehensive assessment of PINK1 variants in Parkinson’s disease. Neurobiology Of Aging. 2020 Jul;91:168.e1–168.e5. doi: 10.1016/j.neurobiolaging.2020.03.003 PMC7236133 32249012

[49] Zhu W, Huang X, Yoon E, et al. Heterozygous PRKN mutations are common but do not increase the risk of Parkinson’s disease. Brain. 2022 Jun 30;145(6):2077–2091. doi: 10.1093/brain/awab456 35640906 PMC9423714

[50] Yu E, Rudakou U, Krohn L, et al. Analysis of heterozygous PRKN variants and copy-number variations in Parkinson’s disease. Mov Disord. 2021 Jan;36(1):178–187. doi: 10.1002/mds.28299 32970363

[51] Lubbe SJ, Bustos BI, Hu J, et al. Assessing the relationship between monoallelic PRKN mutations and Parkinson’s risk. Hum Mol Genet. 2021 Mar 25;30(1):78–86. doi: 10.1093/hmg/ddaa273 33448283 PMC8033143

[52] Puschmann A, Fiesel FC, Caulfield TR, et al. Heterozygous PINK1 p.G411S increases risk of Parkinson’s disease via a dominant-negative mechanism. Brain. 2017 Jan;140(1):98–117. doi: 10.1093/brain/aww261 27807026 PMC5379862

[53] Soutar MPM, Melandri D, O’Callaghan B, et al. Regulation of mitophagy by the NSL complex underlies genetic risk for Parkinson’s disease at 16q11.2 and MAPT H1 loci. Brain. 2022 Dec 19;145(12):4349–4367. doi: 10.1093/brain/awac325 36074904 PMC9762952

[54] Wu Y, Fu Y, Guo J, et al. Single-molecule immunoassay technology: recent advances. Talanta. 2023 Dec 1;265:124903. doi: 10.1016/j.talanta.2023.124903 37418954

[55] Hwang J, Banerjee M, Venable AS, et al. Quantitation of low abundant soluble biomarkers using high sensitivity single molecule counting technology. Methods. 2019 Apr 1;158:69–76. doi: 10.1016/j.ymeth.2018.10.018 30394294

[56] Fiesel FC, Fricova D, Hayes CS, et al. Substitution of PINK1 Gly411 modulates substrate receptivity and turnover. Autophagy. 2022 Dec;5(6):1–22. doi: 10.1080/15548627.2022.2151294 PMC10262784 36469690

[57] Braak H, Del Tredici K, Rub U, et al. Staging of brain pathology related to sporadic Parkinson’s disease. Neurobiol Aging. 2003 Mar-Apr;24(2):197–211. doi: 10.1016/S0197-4580(02)00065-9 12498954

[58] Thal DR, Rub U, Orantes M, et al. Phases of Aβ-deposition in the human brain and its relevance for the development of AD. Neurology. 2002 Jun 25;58(12):1791–1800. doi: 10.1212/WNL.58.12.1791 12084879

[59] Armbruster DA, Pry T. Limit of blank, limit of detection and limit of quantitation. Clin Biochem Rev. 2008 Aug;29 Suppl 1(Suppl 1):S49–52. 18852857 PMC2556583

