# Supplementary material for: Development and validation of a sensitive sandwich ELISA against human PINK1
Source: Autophagy. 2025 Feb 6;21(5):1144–59. doi: 10.1080/15548627.2025.2457915 (PMC12013435; doi:10.1080/15548627.2025.2457915)
Supplement: Baninameh_SUPPL_R3.docx [file KAUP_A_2457915_SM7865.docx]

**SUPPLEMENTARY MATERIAL**

# Development and validation of a sensitive sandwich ELISA against human PINK1

Zahra Baninameh^1^, Jens O Watzlawik^1^, Bernardo A Bustillos^1^, Gabriella Fiorino^1^, Tingxiang Yan^1^, Szymon L. Lewicki^1^, Haonan Zhang^1^, Dennis W Dickson^1,2^, Joanna Siuda^3^, Zbigniew K Wszolek^4^, Wolfdieter Springer^1,2^, and Fabienne C Fiesel^1,2,*^

^1^ Department of Neuroscience, Mayo Clinic, 4500 San Pablo Road, Jacksonville, FL 32224

^2^ Neuroscience PhD Program, Mayo Graduate School of Biomedical Sciences, Mayo Clinic, Jacksonville, FL 32224, USA.

^3^ Department of Neurology, Faculty of Medical Sciences in Katowice, Medical University of Silesia, Katowice 40-055, Poland

^4^ Department of Neurology, Mayo Clinic, 4500 San Pablo Road, Jacksonville, FL 32224

^*^ Corresponding author

Correspondence should be addressed to:

Fabienne C Fiesel, PhD; email: [Fabienne.Fiesel@mayo.edu](mailto:Fabienne.Fiesel@mayo.edu)

## Table S1. Raw data of PINK1 MSD sandwich antibody combinations.

|  |  | |  | coating antibody | | | | | | | | | | |
| --- | --- | --- | --- | --- | --- | --- | --- | --- | --- | --- | --- | --- | --- | --- |
|  | |  | samples | **1** | **3** | **4** | **10** | **11** | **12** | **13** | **14** | **16** | **19** | **20** |
| detecting antibody | | **1** | WT |  |  |  | 1,259 | 1,353 | 282 | 1,052 | 1,277 | 1,026 | 603 | 336 |
|  |  |  | WT 8 h |  |  |  | 5,069 | 3,988 | 1,343 | 2,912 | 3,881 | 3,617 | 1,752 | 1,112 |
|  |  |  | KO 8 h |  |  |  | 993 | 1,180 | 244 | 1,040 | 1,199 | 771 | 611 | 327 |
|  |  |  | Blank |  |  |  | 1,446 | 1,648 | 772 | 1,587 | 1,783 | 1,181 | 795 | 439 |
|  |  | **3** | WT |  |  |  | 877 | 613 | 226 | 698 | 470 | 296 | 294 | 180 |
|  |  |  | WT 8 h |  |  |  | 1,132 | 839 | 377 | 883 | 645 | 494 | 333 | 230 |
|  |  |  | KO 8 h |  |  |  | 809 | 636 | 227 | 695 | 469 | 275 | 304 | 195 |
|  |  |  | Blank |  |  |  | 665 | 683 | 216 | 700 | 457 | 247 | 348 | 298 |
|  |  | **4** | WT |  |  |  | 1,287 | 1,226 | 319 | 1,244 | 1,424 | 787 | 305 | 229 |
|  |  |  | WT 8 h |  |  |  | 1,721 | 1,531 | 531 | 1,431 | 1,822 | 1,064 | 418 | 304 |
|  |  |  | KO 8 h |  |  |  | 1,337 | 1,112 | 312 | 1,183 | 1,457 | 820 | 327 | 236 |
|  |  |  | Blank |  |  |  | 1,001 | 821 | 237 | 830 | 901 | 386 | 383 | 340 |
|  |  | **10** | WT | 777 | 782 | 296 |  |  |  |  |  |  | 187 | 145 |
|  |  |  | WT 8 h | 3,801 | 1,792 | 1,104 |  |  |  |  |  |  | 440 | 326 |
|  |  |  | KO 8 h | 421 | 762 | 234 |  |  |  |  |  |  | 174 | 147 |
|  |  |  | Blank | 246 | 298 | 142 |  |  |  |  |  |  | 235 | 199 |
|  |  | **11** | WT | 471 | 227 | 181 |  |  |  |  |  |  | 196 | 124 |
|  |  |  | WT 8 h | 2,384 | 783 | 819 |  |  |  |  |  |  | 345 | 205 |
|  |  |  | KO 8 h | 256 | 190 | 137 |  |  |  |  |  |  | 192 | 123 |
|  |  |  | Blank | 207 | 170 | 143 |  |  |  |  |  |  | 249 | 167 |
|  |  | **12** | WT | 448 | 226 | 214 |  |  |  |  |  |  | 135 | 93 |
|  |  |  | WT 8 h | 1,884 | 635 | 677 |  |  |  |  |  |  | 225 | 163 |
|  |  |  | KO 8 h | 298 | 196 | 183 |  |  |  |  |  |  | 131 | 88 |
|  |  |  | Blank | 243 | 172 | 162 |  |  |  |  |  |  | 157 | 115 |
|  |  | **13** | WT | 229 | 197 | 153 |  |  |  |  |  |  | 263 | 189 |
|  |  |  | WT 8 h | 283 | 213 | 181 |  |  |  |  |  |  | 266 | 192 |
|  |  |  | KO 8 h | 231 | 207 | 150 |  |  |  |  |  |  | 277 | 192 |
|  |  |  | Blank | 197 | 180 | 145 |  |  |  |  |  |  | 333 | 299 |
|  |  | **14** | WT | 205 | 189 | 141 |  |  |  |  |  |  | 127 | 115 |
|  |  |  | WT 8 h | 246 | 200 | 157 |  |  |  |  |  |  | 126 | 117 |
|  |  |  | KO 8 h | 204 | 192 | 140 |  |  |  |  |  |  | 131 | 111 |
|  |  |  | Blank | 189 | 168 | 136 |  |  |  |  |  |  | 172 | 150 |
|  |  | **16** | WT | 515 | 177 | 162 |  |  |  |  |  |  | 93 | 104 |
|  |  |  | WT 8 h | 1,915 | 650 | 657 |  |  |  |  |  |  | 257 | 250 |
|  |  |  | KO 8 h | 342 | 149 | 127 |  |  |  |  |  |  | 83 | 92 |
|  |  |  | Blank | 301 | 143 | 127 |  |  |  |  |  |  | 86 | 96 |
|  |  | **19** | WT | 11,059 | 285 | 266 | 72,250 | 26,144 | 16,865 | 21,048 | 143,273 | 172,198 |  |  |
|  |  |  | WT 8 h | 10,813 | 354 | 325 | 72,459 | 27,958 | 16,779 | 20,615 | 145,267 | 183,857 |  |  |
|  |  |  | KO 8 h | 10,041 | 302 | 270 | 73,453 | 28,923 | 16,947 | 21,388 | 144,624 | 178,805 |  |  |
|  |  |  | Blank | 9,642 | 212 | 177 | 72,782 | 29,596 | 17,480 | 21,755 | 138,146 | 188,927 |  |  |
|  |  | **20** | WT | 249 | 323 | 320 | 97,724 | 3,983 | 17,873 | 211 | 114,749 | 170,272 |  |  |
|  |  |  | WT 8 h | 270 | 335 | 347 | 101,524 | 3,844 | 18,031 | 250 | 117,662 | 175,827 |  |  |
|  |  |  | KO 8 h | 237 | 317 | 323 | 102,660 | 3,948 | 18,011 | 188 | 122,638 | 171,059 |  |  |
|  |  |  | Blank | 228 | 361 | 359 | 102,016 | 3,839 | 20,219 | 154 | 122,044 | 165,990 |  |  |

Shown is the average of the ECL signal data from duplicate samples that were run for each possible antibody combination, as indicated. Antibodies are numbered and color-coded for their host species as in Table 1. Because the sandwich ELISA relies on a species-specific secondary sulfo-tag-coupled antibody, same species antibody combinations were not tested.
